# Transition from Persistent to Anti-Persistent Correlations in Postural Sway Indicates Velocity-Based Control

**DOI:** 10.1371/journal.pcbi.1001089

**Published:** 2011-02-24

**Authors:** Didier Delignières, Kjerstin Torre, Pierre-Louis Bernard

**Affiliations:** EA 2991 Movement To Health, Montpellier-1 University, Euromov, Montpellier, France; University College London, United Kingdom

## Abstract

The displacement of the center-of-pressure (COP) during quiet stance has often been accounted for by the control of COP position dynamics. In this paper, we discuss the conclusions drawn from previous analyses of COP dynamics using fractal-related methods. On the basis of some methodological clarification and the analysis of experimental data using stabilogram diffusion analysis, detrended fluctuation analysis, and an improved version of spectral analysis, we show that COP velocity is typically bounded between upper and lower limits. We argue that the hypothesis of an intermittent velocity-based control of posture is more relevant than position-based control. A simple model for COP velocity dynamics, based on a bounded correlated random walk, reproduces the main statistical signatures evidenced in the experimental series. The implications of these results are discussed.

## Author Summary

Postural control during quiet standing is usually conceived of as the control of position: when position goes beyond a given threshold, corrective mechanisms are engaged to restore equilibrium. In this paper, we question this conception and show that postural control is based on an intermittent control of velocity, with a reversal in its dynamics when the absolute value of velocity reaches a given threshold. This hypothesis presents some counterintuitive implications. Notably, it means that the active control or correction processes do not intervene at the periphery of postural sways, as generally assumed. According to our findings, control occurs in the central region of the posturogram, where velocity reaches its maximal absolute values. The present study suggests new variables of interest in the study of postural control, especially the maximal absolute velocity of the center-of-pressure, which could describe and predict postural disorders.

## Introduction

Postural control during quiet stance has mainly been studied at the macroscopic behavioral level by assessing the displacement of the center-of-pressure (COP). The highly complex dynamics of COP has often been reduced to the magnitude of its variability and examined comparatively between different conditions of stance (*e.g.*, open versus closed eyes, [1]), various ages [2], and healthy populations versus neurodegenerative patients [3], [4]. Newell et al. [5], however, argued that such descriptive statistics, based on the averaging of COP measures over time, could conceal the control principles that underlie the observed postural dynamics and emphasized the value of a time series approach.

In the past few decades, researchers have studied COP trajectory using a variety of nonlinear time series analyses. They have assessed, for instance, the predictability of the COP trajectory using recurrence quantification analysis [6], [7], [8], the chaotic nature of postural sway through the Lyapunov exponent [9]–[11], the irregularity of fluctuations using sample entropy [12]–[14], and the structure of serial (long-range) correlations in the COP signals using various fractal methods [15]–[19].

Such time series analyses are based on the idea that the temporal structure of COP fluctuations captures the organization of the complex, nonlinear, and dynamical “control” processes of the postural system. While these approaches have provided original and interesting insights into the processes underlying COP dynamics, one cannot help but note that the results have sometimes been contradictory and often not directly comparable. The main issue with such methods is that while they may require specific preconditions to be properly applied to the time series, they always give *some* result which may mislead further interpretations. In the present paper, we develop one particular example of how fuzziness in time series analysis can actually lead to a choice between two opposite conclusions about the control processes underlying COP dynamics.

Based on the general assumption that COP dynamics can be represented by the family of stochastic processes, Collins and De Luca [16], [17] proposed to characterize the correlations contained in experimental COP series using stabilogram diffusion analysis (SDA). Note that in the time series framework, a (serial) positive correlation signifies that an increasing trend in the past is likely to be followed by an increasing trend in the future. The series is said to be persistent. Conversely, a negative correlation signifies that an increasing trend in the past is likely to be followed by a decreasing trend. The series is then said to be anti-persistent.

The results of Collins and De Luca [16], [17] suggested that COP position series were positively correlated in the short term (*i.e.*, over short observation times) but negatively correlated in the long term. The transition from persistent to anti-persistent correlation regimes over different time scales is known as a “cross-over phenomenon” [20]. Cross-over is typically related to the fact that variables are bounded within given limits [20]. Such bounding effects are essential as they suggest that a type of control, whether direct or indirect, is exerted on the variable. Following this line of thought, Collins and De Luca [16], [17] supported the idea that postural control may be position-based. The authors argued that postural sway displacements “are left unchecked by the postural control system until they exceed some systematic threshold” [16, p. 317] and that “the presence of longer-range negative correlations in the COP data suggests that closed-loop mechanisms are utilized over long-term intervals of time and large displacements” [17, p.767].

In this present paper, we clarify some of the methodological issues related to the use of fractal analysis in the studies by Collins and De Luca [16], [17]. These explanations provide support for the idea that the control of postural sway is velocity-based instead of position-based. We confirm this idea with the results obtained from the analysis of experimental data and then propose a very simple phenomenological model in accordance with our present considerations.

### The key issue in Collins and De Luca (1993)'s methodology and fractal-related analyses

Two common methods for characterizing the serial correlation properties of postural data are stabilogram diffusion analysis (SDA) [16], [17], [21], [22] and detrended fluctuation analysis (DFA) [14], [18], [23], [24]. These methods share the same theoretical foundation and are actually quite similar. To clearly set out the methodological issues and related interpretations of Collins and De Luca's approach, we need to bring these two methods within the general framework of fractal processes.

**Figure 1:**
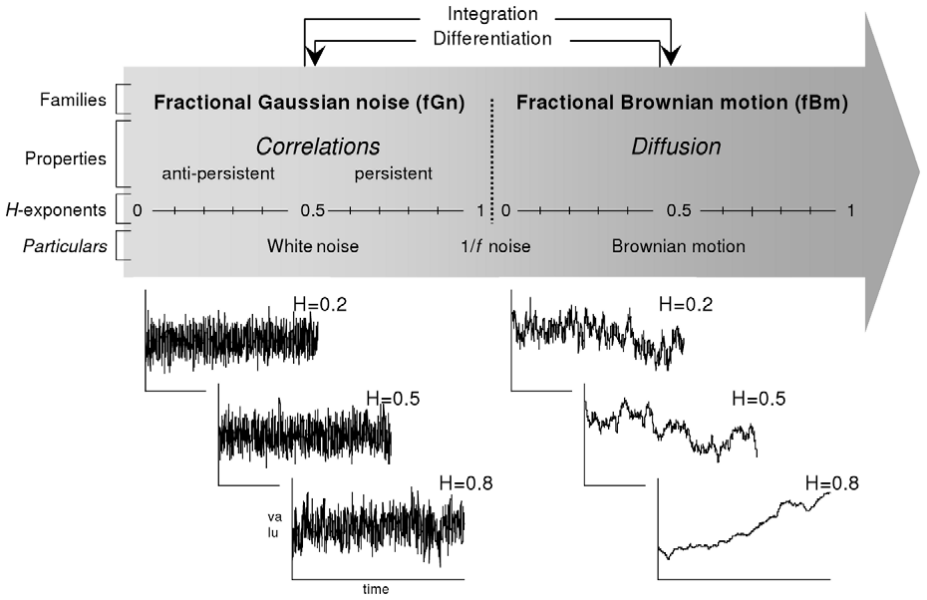
Fractional Gaussian noise and fractional Brownian motion. Representation of the continuum of fractal processes, with: the two families of fractional Gaussian noise and fractional Brownian motion, the typical correlation and diffusion properties characterizing the two types of processes, and the associated *H* exponents.

Fractal processes can be categorized in two families: *fractional Gaussian noise* (fGn), which represents stationary series with a constant mean and variance, and *fractional Brownian motions* (fBm), which are non-stationary series with time-dependent variance (Figure 1). By definition, the variance of displacement for a fBm is a power function of the time over which this displacement is observed, so that it obeys the following scaling law [25]:
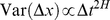
(1)or, equivalently
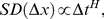
(2)where *H* ranges between 0 and 1. This scaling law expresses the so-called *diffusion property* specific to fBm processes, whose characteristics depend on the exponent *H*. The higher the *H*, the more diffusive the fBm will be. Intuitively, one could consider that diffusion represents the probabilistic dispersion of the process, relative to the initial position, after a given time interval Δ*t*, and for multiple replications of the process. Note that the fBm family is centered on the particular case *H* = 0.5, which corresponds to ordinary Brownian motion for which variance is proportional to the expended time [26].

fBm and fGn processes are related by integration and differentiation, and they are characterized by the same *H* exponents: the differentiation of a fBm gives the corresponding fGn and, conversely, the integration of a fGn is the corresponding fBm (see Figure 1). In contrast to the fBm, the diffusion property is not present in a fGn. Instead, one can classify the fGn with respect to the correlation properties of the series. For *H*>0.5, the series contain persistent correlations, and for *H*<0.5, fGn series are anti-persistent. One can intuitively understand that persistent correlation in the fGn leads to strong diffusion in the corresponding fBm, and vice versa. Additional considerations about the fGn/fBm model can be found in previous papers [27], [28].

With respect to our present methodological issue, the correspondence between fGn and fBm implies that one can assess the diffusion properties of a fBm in order to infer the correlation structure of the corresponding fGn. In other words, if one wants to assess the correlation properties of a fGn (or stationary series) using methods for working on diffusion properties, the series under study needs to be integrated prior to analysis.

Both SDA and DFA work on the diffusion properties of series and are based on the scaling law of Equation 1. Basically, SDA computes the mean variance of COP displacement for a given time interval length Δ*t*. This calculation is repeated as a function of increasing values of Δ*t* (see Methods section for details). According to Equation 1, the slope of the resulting bi-logarithmic *diffusion plot*, expressing variance as a function of time interval, is expected to be 2*H*, ranging between 0 and 2. Thus, a slope equal to 1 represents a boundary value in the SDA-diffusion plots: a slope<1 indicates anti-persistent correlations while a slope>1 indicates persistent correlations in the differenced series.

DFA is also based on the assessment of variability within intervals of varying lengths. However, the DFA algorithm differs slightly from the SDA algorithm and especially in a first step the series is integrated. The mean standard deviation of this integrated series is then determined as a function of the interval lengths (see Methods section for details). Because of the integration step in the analysis, this method directly assesses the correlation properties of the analyzed series, and not those of the differenced series, as for SDA. The slope of the resulting diffusion plot in bi-logarithmic coordinates, according to Equation 2, is expected to be *H*, ranging between 0 and 1, if the series analyzed is a fGn, and *H*+1, ranging between 1 and 2, if the series is a fBm. Thus, 0.5 is the boundary value for the DFA diffusion plots: the analyzed series are stationary and contain anti-persistent correlations for slopes<0.5 (for fGn series) while they contain persistent correlations for slopes>0.5.

**Figure 2:**
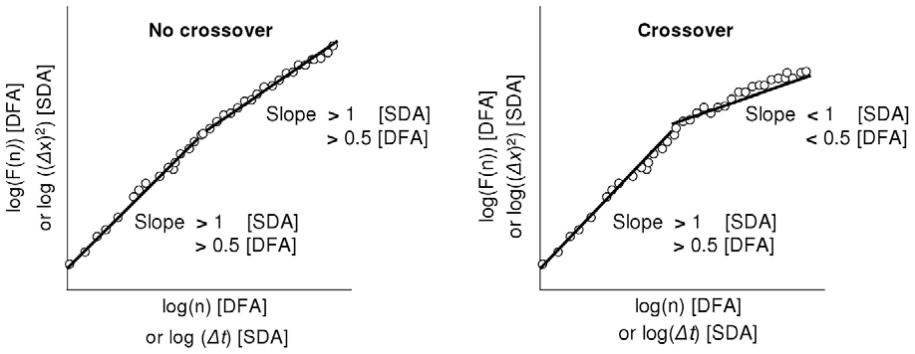
Graphical signatures of cross-over. Schematic representation of the typical log-log diffusion plots resulting from SDA and DFA. This figure illustrates how the cross-over phenomenon can be detected using diffusion analysis.

Figure 2 shows a schematic representation of typical diffusion plots obtained with SDA and DFA. Obtaining an inflection point in the diffusion plot (Figure 2, right graph), with slope values changing from greater than to less than the above-cited boundary values, indicates the so-called *cross-over phenomenon*. It shows a transition from persistent correlations on short observation scales to anti-persistent correlations on longer observation scales in the corresponding differenced series, thereby indicating that the latter is bounded within given limits [20]: the bounded variable derives (*i.e.*, is positively correlated) until reaching a given limit value. At this point, the fluctuations reverse in direction (*i.e.*, the series becomes negatively correlated). Such bounding suggests that the variable concerned is (in)directly controlled. Note that bounded series are obviously stationary (at least in the long term), but cannot be considered as genuine fGn.

Now, the crucial difference between DFA and SDA is that the DFA algorithm includes the integration of the analyzed series whereas the SDA does not. In a previous paper [18], we highlighted this shortcoming in Collins and De Luca's approach, but we failed to capture a major implication of the obtained results. By applying SDA to COP position series and observing a cross-over, Collins and De Luca [16] argued that postural control was position-based. However, since evidencing the cross-over phenomenon in the SDA diffusion plot of a given series signifies that the *differenced* series is bounded, we argue that the authors' conclusion should have applied to COP velocity. In other words, we suggest that the control of postural sway is velocity-based instead of position-based. To test this assumption, we analyzed experimental postural data using SDA, DFA, and spectral analysis as a complement.

## Results

Twenty-six participants were asked to maintain quiet stance on a force platform. The position of the COP was recorded as time series, with a sampling frequency of 40 Hz (see Methods section for details).

**Figure 3:**
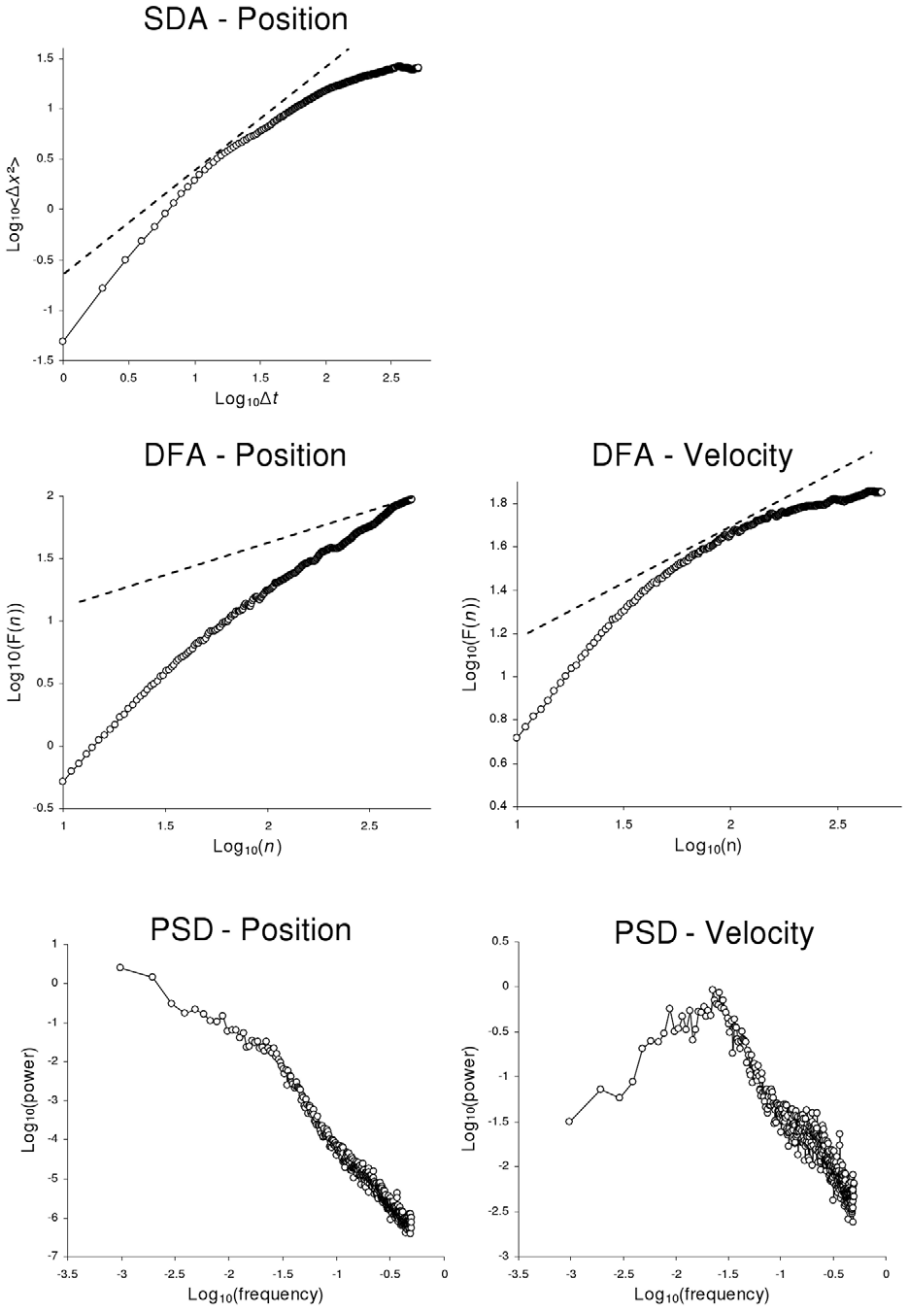
Mean graphical results for experimental series. Average log-log diffusion plots obtained from SDA and DFA, and log-log power spectra on the COP position and velocity data (ML axis) collected during quiet standing. The dashed lines in the upper (SDA) and middle graphs (DFA) represent the boundary slopes between persistent and anti-persistent correlation (slope = 1.0 for SDA, and 0.5 for DFA, see text for details). The SDA shows a cross-over phenomenon when applied to position series while both the DFA and PSD analyses show a cross-over in velocity series.

We first applied SDA on position series, following the procedure proposed by Collins and De Luca [16]. SDA diffusion plots exhibited the two typical correlation regimes, with persistent correlations over the short term and anti-persistent correlations over the long term, indicating a cross-over phenomenon (Figure 3, upper panel).

We then applied DFA to COP position and velocity series. For position series, the DFA diffusion plot revealed persistent correlations over both short and long terms. In other words, DFA did not evidence any cross-over when applied to COP position series. When applied to velocity series, however, the DFA diffusion plot showed a cross-over, with positive correlations over the short term and negative correlations over the long term (Figure 3, middle panels).

We then applied spectral analysis as a complement to the above methods. This method is likely to provide an immediate and visually salient representation of the cross-over phenomenon, with positive slopes in the log-log power spectrum indicating anti-persistence and negative slopes indicating persistence. Thus, the cross-over is expected to be revealed by a positive slope in low frequencies and a clear inflection toward a negative slope in high frequencies. Spectral analysis confirmed the results evidenced by DFA: the cross-over phenomenon was obtained only with velocity series, but not with position series (Figure 3, bottom panels).

**Figure 4:**
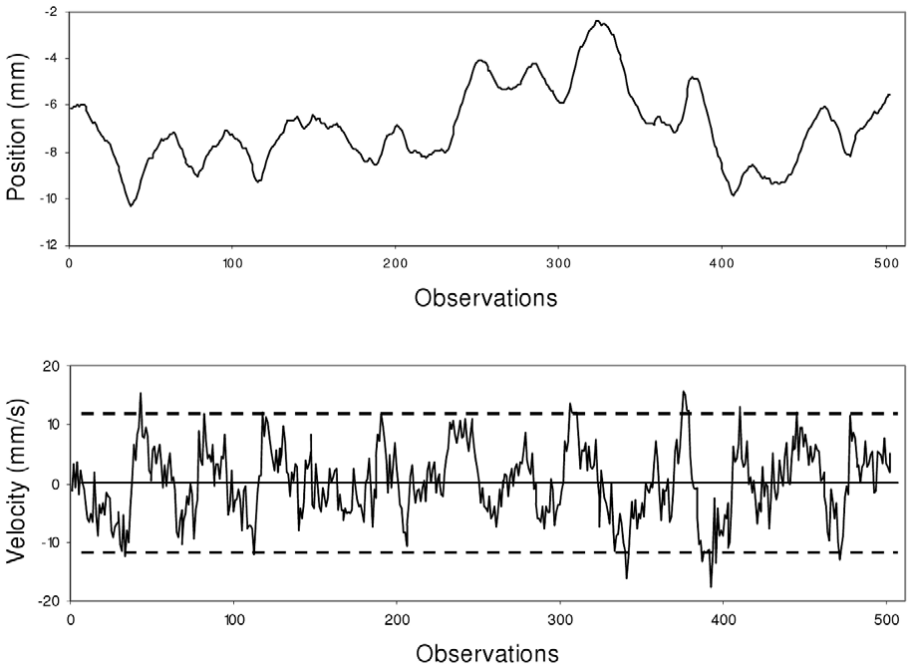
Representative examples of empirical series of COP position (top) and velocity (bottom). Series from participant #10, ML axis.

These results clearly showed that bounding essentially affects COP velocity and not COP position, and one can thus assume that the COP trajectory is the consequence of velocity-based control. In concrete terms, the evidenced bounding means that COP velocity evolves between two (upper and lower) limit values. Its evolution from one limit to the other looks similar to a fractional Brownian motion, yielding the persistent correlations evidenced in the short term. The long-term evolution of COP velocity is characterized by a quite systematic to and fro motion within the range defined by the upper and lower limits; these systematic reversals yield the anti-persistent correlations observed in the long term. Figure 4 illustrates this specific dynamics.

On the basis of these results, we propose a very simple model for COP velocity dynamics in order to determine whether a simple bounding control of velocity would generate the complex trajectories observed in COP. This model accounts for velocity dynamics using a first-order autoregressive process (see Methods section for details):
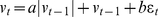
(3)where *v_t_* represents velocity at time *t*, *a* is a constant, *ε_t_* is a white noise process with zero mean and unit variance, and *b* is a constant representing the strength of the noise term. The long-term anti-persistent dynamics of COP velocity is accounted for by reversing the sign of *a* each time that the absolute velocity value exceeds a given threshold *T*.

**Figure 5:**
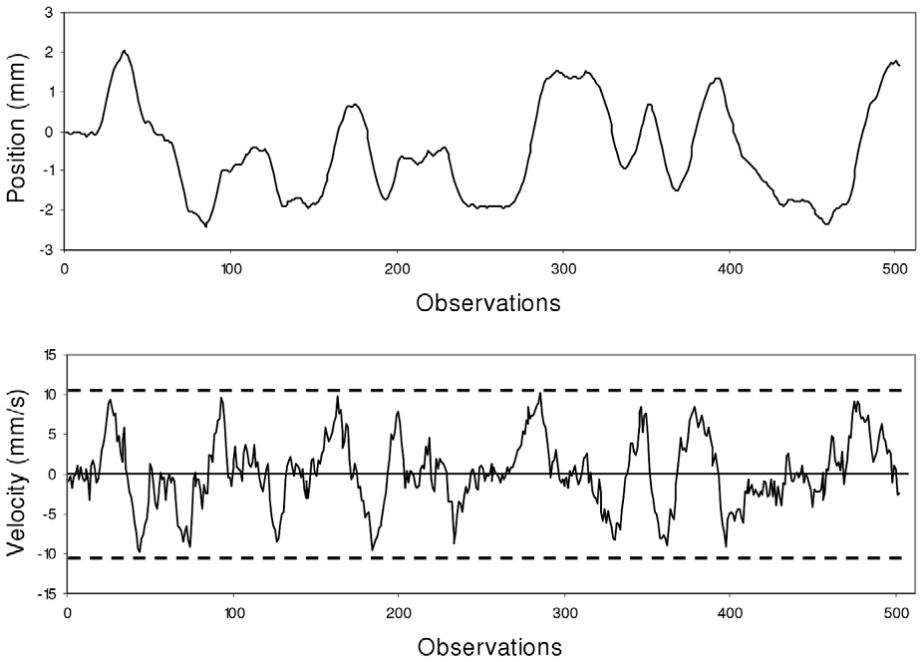
Representative examples of simulated series of COP position (top) and velocity (bottom).

**Figure 6:**
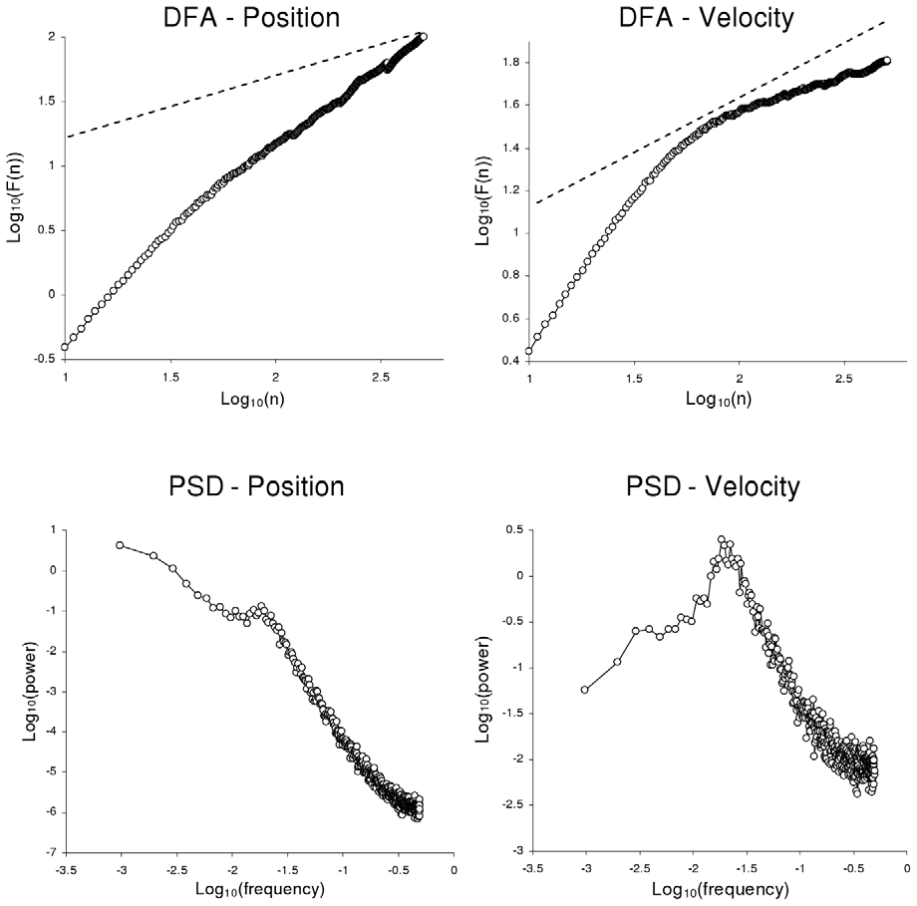
Mean graphical results for simulated series. Average log-log diffusion plots and power spectra obtained from DFA and PSD with simulated position and velocity series. These graphs are based on point-by-point averaging of the results obtained from 26 randomly selected simulated series. The dashed line in the upper plots (DFA) represents the slope of 0.5, corresponding to the boundary between persistent and anti-persistent correlation.

Figure 5 shows an example of the simulated series of position and velocity produced by the model. When the analyses previously used for the experimental series were applied to these simulated series, the model was able to account for the main statistical signatures observed experimentally: DFA and spectral analysis revealed a cross-over for velocity series, but not for position series (see Figure 6).

## Discussion

Let us briefly summarize the rationale for the present study and its main results. Collins and De Luca [16], [17] applied the SDA method to position series and showed a cross-over; they accounted for this finding by proposing that the postural control system prevents COP displacement from exceeding given boundaries. We hypothesized, however, that since SDA does not include an integration step, this result actually revealed a bounding of COP velocity instead of COP position. We thus argued that the cross-over obtained by Collins and De Luca should be interpreted in terms of a velocity-based control instead of a position-based control of posture.

In order to test this hypothesis, we analyzed experimental COP position and velocity data using three different methods: SDA, DFA, and spectral analysis. The DFA algorithm includes a preliminary integration process and thus allows detection of a cross-over in the analyzed series. Spectral analysis also reveals cross-over in a straightforward way. Our results showed that SDA replicated (qualitatively) the earlier results of Collins and De Luca: the diffusion plot of position series showed a cross-over. On the other hand, both DFA and spectral analysis evidenced a cross-over in velocity but not in position series. These results clearly support the hypothesis that bounding affects primarily COP velocity.

This two-scale dynamics suggests that an intermittent control of velocity underlies the COP trajectory, reversing its dynamics when the absolute value of velocity reaches a given threshold. Note that this hypothesis could be conceived as a velocity-based analog of the two-regime model proposed by Collins and De Luca [16]. Our results indeed suggest that sway is left unchecked until a threshold in velocity is reached. Obviously, we are not arguing that COP velocity is directly controlled during upright stance. Balance is maintained by control of the center-of-mass motion, and the COP trajectory is only a macroscopic outcome reflecting some aspects of the underlying control processes. That said, our statistical results nevertheless suggest that postural control is more likely to be velocity-based than position-based. This hypothesis is consistent with the theoretical predictions of the *noisy-computation* model proposed by Kiemel et al. [29] and the subsequent experiment performed by Jeka et al. [30], suggesting the crucial role of velocity information for postural stability. Velocity information about the center of mass dynamics is provided by two sensory modalities: vision and proprioception from the feet/ankles [29], [30]. In their experiment, Jeka et al. [30] degraded the velocity information by removing/attenuating the sensory information from the visual and proprioceptive systems. They showed that a deficit in information about velocity, rather than position or acceleration, affected postural sway and they concluded that velocity information was the most accurate form of sensory information to stabilize posture.

This hypothesis presents some counterintuitive implications. Notably, it means that the active control or correction processes do not intervene at the periphery of the COP trajectory, *i.e.*, when postural sway exceeds a given surface, as generally assumed (*e.g.*, [16], [17]). According to our present findings, control instead intervenes in the central region of the stabilogram, where COP velocity reaches its maximal absolute values.

Finally, while the dynamics of the COP trajectory has usually been described as very complex, our results suggested a quite simple model of velocity dynamics. This model reproduced the main statistical properties evidenced experimentally. A number of models have been proposed to account for COP dynamics during quiet stance [29], [31]–[35]. These models are based on inverted pendulum dynamics [31], [35] or more formal dynamical equations [29], [32]–[34] and include various ingredients to account for neurophysiology-relevant processes such as control torques [35], time delays [31], [35], and stochastic perturbation [33]. All these models were proven to efficiently mimic the statistical characteristics of COP dynamics. The originality of the present model is that it directly accounts for COP velocity instead of position, as opposed to previous models. Note, however, that this model is not intended to represent the actual processes involved in postural control. Its aim is simply to show that the bounding of velocity evidenced in the analysis of empirical series is sufficient for mimicking the main features of COP dynamics. Even if this model lacks physiological realism, it offers new ways of thinking about modeling, as a complement to previous proposals.

**Figure 7:**
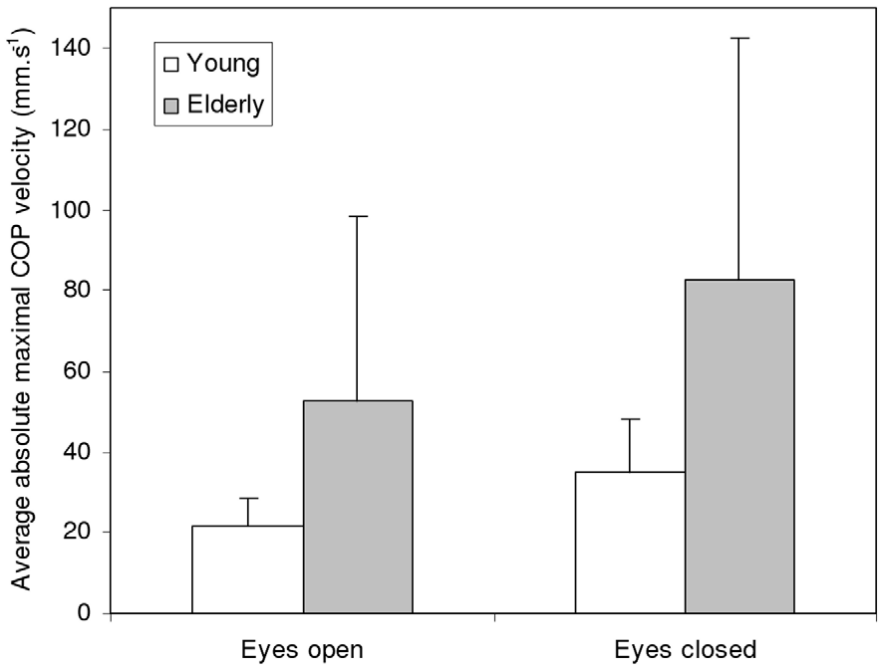
Effects of age and vision on the average absolute maximal velocity of the COP. Results are given for the AP axis.

The present study suggests new variables of interest in the study of postural control. Beyond the signatures of serial correlations addressed here, the determinants of the threshold that bounds the dynamics of velocity may be of particular interest. The value of this threshold can be empirically estimated by computing the *average absolute maximal velocity* (AAMV) of the COP. This computation can be easily done from velocity series by extracting the maximum and minimum values of the series within non-overlapping windows. The length of these windows should be chosen to ensure the collection of at least one maximum and one minimum (*e.g.*, 2 sec). The absolute values of these extrema are then averaged. In a preliminary investigation, we computed the AAMV in two groups of participants differing in age (young, *N* = 26, mean age 19.3±2.1; elderly, *N* = 25, mean age 76.1±5.8). Data were collected in two conditions of vision (eyes open and eyes closed). We obtained a significant effect of the first factor (F(1,51) = 13.86, p<.000), indicating that AAMV increases with age. The effect of vision was also significant (F(1,51) = 56.21; p<.000), showing an increase in AAMV in the absence of vision. The interaction effect (F(1,51) = 8.22, F<0.007) indicated that the effect of the absence of vision was greater in the elderly (see Figure 7). These results suggest the potential interest of this variable for analyzing the effect of task modalities and individual characteristics on the dynamics of COP. In particular, AAMV may be a predictor of fall risk in the elderly.

This study shows how sophisticated methods for the assessment of the complex properties of experimental time series must be used with much caution and regard to their theoretical and methodological foundations. Perhaps more than for other more “classical” analysis, the conclusions drawn from such methods are directly dependent on the specific properties of the algorithms and procedures implemented.

## Methods

### Ethics statement

This study was conducted according to the principles expressed in the Declaration of Helsinki. The study was approved by the Institutional Review Board of the University of Montpellier 1. All patients provided written informed consent for the collection of samples and subsequent analysis.

### Experimental setup

Twenty-six male volunteers (19.3 yrs±2.1) took part in the experiment. The participants were asked to maintain quiet stance on a force platform (Medicapteurs “40 Hz/16b”) of 530 mm×460 mm×35 mm, equipped with three pressure gauges. Participants held their arms alongside their body and focused on a visual reference mark fixed 90 cm in front of them. The feet were oriented with an angle of 15° from the sagittal midline, and the heels were positioned 4 cm apart. The participants had a 30-s familiarization period before testing began.

The vertical ground reaction forces were recorded using a 12-bit A/D converter, with a sampling frequency of 40 Hz. The system was linked to Medicapteurs Winposture2000 software, providing COP series on the anteroposterior (AP) and mediolateral (ML) axes. The duration of each recording was 25.6 s, in order to obtain time series with 1024 points. The collected series were filtered by a low-pass filter, with a cut-off frequency of 8 Hz.

### Data analysis

We analyzed COP position and COP velocity data in the ML and AP axes. The velocity series were obtained by differentiating the position series. Note that the velocity series were not further filtered after differentiation. First, we applied SDA on the position series, following the procedure proposed by Collins and De Luca [16]. This method consists of computing the square of the displacement (Δ*x*)^2^ within all pairs of points separated by a time interval Δ*t*. This computation is repeated for increasing values of Δ*t*. The resulting diffusion plot represents the mean squared displacements against the time intervals Δ*t*, in bi-logarithmic coordinates. We considered time intervals ranging between Δ*t* = 1 and Δ*t* = 341, (*i.e.*, between 25 ms and 8525 ms). Note that the highest values still allow the estimation of (Δ*x*)^2^ to be based on three non-overlapping intervals.

Second, we applied DFA to the COP position and velocity series. DFA includes a series of operations: First the analyzed series *x*(*t*) is integrated, by computing for each *t* the accumulated departure from the mean of the whole series:
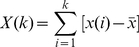
(4)


The integrated series is then divided into non-overlapping intervals of length *n*. In each interval, the least squares regression line (representing the local trend within the interval) is fitted to the data. The series *X(k)* is then locally detrended by subtracting the theoretical values *X_n_(k)* given by the regression. Finally, for each interval length *n*, the characteristic size of the fluctuation for this integrated and detrended series is given by:
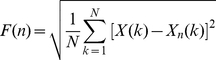
(5)


Because DFA needs a minimal number of points to compute the standard deviation within each interval, we considered intervals from *n* = 10 to 512 [27].

In complement to the above methods in the temporal domain, we applied power spectral density (PSD) analysis, which was likely to provide an immediate and visually salient representation of the cross-over phenomenon. PSD allows assessing serial correlation in a signal because the scaling law of Equation 2 can be expressed as follows in the frequency domain:
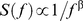
(6)where *f* is the frequency and *S(f)* the corresponding squared amplitude. This power relationship is revealed in the bi-logarithmic power spectrum by a linear regression of slope -β. Thus, a positive slope indicates anti-persistent correlations and a negative slope indicates persistent correlations (see [27], [28] for a deeper presentation of the use of PSD for fractal analysis). A separate assessment of serial correlation over the short and long terms can then be obtained by fitting separate linear regression lines to the high-frequency and the low-frequency regions of the log-log spectrum, respectively.

Preprocessing operations were used before the application of the fast Fourier transform algorithm: First the mean of the series was subtracted from each value, and then a parabolic window was applied: each value in the series was multiplied by the following function:
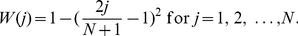
(7)


Third, bridge detrending was performed by subtracting from the data the line connecting the first and last points of the series. These preprocessing operations have been recommended by Eke et al. [28] to improve the assessment of correlation properties with PSD.

In order to avoid any bias due to the logarithmic distributions of the points in the diffusion plots and power spectra, we divided the abscissa into intervals of equal lengths (24 intervals of 0.1(log_10_Δ*t*) for SDA, 18 intervals of 0.1(log_10_ *n*) for DFA, and 25 intervals of 0.1(log_10_Δ*f*) for PSD), computed the average points within each interval, and determined the regression slopes over these average points. Finally, for an accurate estimation of the regression slopes in the short-term/high-frequency and long-term/low-frequency regions, we excluded the central part of the plots and performed the regressions on the first and the last parts of the graph (from points 1 to 8 and 17 to 24 for SDA; from points 1 to 6 and 12 to 18 for DFA; from points 1 to 10 and 14 to 25 for PSD). These intervals were chosen after visual inspection of the individual graphs, in order to maintain the inflection point within the central zone in all cases.

These analyses were performed separately on the ML and AP series. To test statistically for the persistence/anti-persistence of serial correlations, we used one-sample *t*-tests to compare the obtained slope with the boundary value of the corresponding method (*i.e.*, 1 for SDA, 0.5 for DFA, and 0 for PSD). In addition, the slopes obtained in the short and long terms, and in the AP and ML directions, were compared using two-factor repeated measures ANOVAs.

The results obtained with the three methods on the COP position and velocity series are summarized in Figure 3 and Table 1. Figure 3 presents the average diffusion plots and power spectra and Table 1 displays the corresponding mean regression slopes.

**Table 1:** Results of time series analyses for COP position and velocity series.

		Position		Velocity	
Method	Slope	AP	ML	AP	ML
SDA	Short-term slope	1.60 (0.19)	1.75 (0.09)	-	-
	Long-term slope	0.48 (0.40)	0.36 (0.33)	-	-
DFA	Short-term slope	1.65 (0.08)	1.70 (0.07)	1.00 (0.17)	1.17 (0.12)
	Long-term slope	1.22 (0.22)	1.00 (0.29)	0.43 (0.12)	0.23 (0.12)
PSD	High-frequency slope	−3.24 (0.39)	−3.32 (0.33)	−1.44 (0.39)	−1.52 (0.33)
	Low-frequency slope	−1.80 (0.50)	−1.60 (0.58)	0.71 (0.54)	1.20 (0.61)

ML: mediolateral direction. AP: anteroposterior direction. Standard deviations are in parentheses.

### Stabilogram diffusion analysis

The results of SDA on the COP position series showed a typical two-regime diffusion plot (Figure 3). The regression slopes were statistically different in the short and long terms (AP: *F*(1,25) = 167.10, *p* = 0.000; ML: *F*(1,25) = 373.38, *p* = 0.000); they indicated highly persistent behavior in the short term (slope>1; AP: *t*(25) = 16.51, p = 0.000; ML: *t*(25) = 43.03, p = 0.000) and anti-persistent correlations (slope<1; AP: *t*(25) = −6.70, p = 0.000; ML: *t*(25) = −9.86, p = 0.000) in the long term. The short-term slope was significantly higher in the ML than in the AP direction (*F*(1,25) = 30.88, *p* = 0.000), but there was no statistical difference between the long-term slopes in ML and AP (*F*(1,25) = 1.97, *p* = 0.173). In sum, SDA showed a cross-over phenomenon when applied to the COP position series.

### Detrended fluctuation analysis

For the position series, the DFA diffusion plot showed a globally positive trend over the short and long terms (Figure 3). Although the slopes were significantly higher in the short term than in the long term (AP: *F*(1,25) = 83.90, *p* = 0.000; ML: *F*(1,25) = 141.53, *p* = 0.000), they indicated persistent correlations for both time scales (slope>0.5; short-term region: AP: *t*(25) = 74.07, *p* = 0.000; ML: *t*(25) = 93.62, *p* = 0.000; long-term region: AP: *t*(25) = 16.57, p = 0.000; ML: *t*(25) = 8.78, *p* = 0.000). Comparison between ML and AP data showed that the slope was higher in the ML than the AP direction for the short-term region (*F*(1,25) = 13.88, *p* = 0.000), and conversely for the long-term region (*F*(1,25) = 9.68, *p* = 0.005). In sum, DFA did not evidence any cross-over phenomenon when applied to the COP position series.

For the velocity series, the DFA diffusion plot showed a much more pronounced inflection between the short- and the long-range regions (see Figure 3). The short-term regression slopes were close to 1, thus indicating persistent serial correlations. In contrast, the slopes in the long-term region were less than 0.5 (AP: *t*(25) = −2.98, p = 0.006; ML: *t*(25) = −11.22, *p* = 0.000), showing the presence of negative correlations. In the short-term region the slopes were higher in the ML than in the AP velocity series (*F*(1,25) = 25.76, *p* = 0.000), and conversely for the long-term region (*F*(1,25) = 45.72, *p* = 0.000). In sum, DFA clearly evidenced a cross-over phenomenon when applied to the COP velocity series.

### Spectral analysis

For the position series, the log-log power spectrum exhibited a globally negative trend (Figure 3). However, the slope was significantly steeper (*i.e.*, more negative) in the high-frequency region than in the low-frequency region (AP: *F*(1,25) = 200.32, *p* = 0.000; ML: *F*(1,25) = 140.23, *p* = 0.000). There was no significant difference between the slopes obtained in the ML and AP directions (low-frequency slopes: *F*(1,25) = 2.84, *p* = 0.104; high-frequency slopes: *F*(1,25) = 0.95, *p* = 0.338).

For the velocity series, the spectral analysis showed two qualitatively different scaling behaviors, with a positive mean slope in the low-frequency region (AP: *t*(25) = 6.65, p = 0.00; ML: *t*(25) = 10.07, *p* = 0.000), and a negative mean slope in the high-frequency region (AP: *t*(25) = −18.73, p = 0.00; ML: *t*(25) = −23.54, *p* = 0.000). In the low-frequency region, the mean slope was lower in the ML than in the AP direction (*F*(1,25) = 5.98, *p* = 0.022), and there was no difference between the AP and ML directions for the high-frequency slopes (*F*(1,25) = 0.95, *p* = 0.339). In sum, the spectral analysis demonstrated a cross-over when applied to the COP velocity series.

### Model

Given these results, we proposed to model the velocity dynamics, considering the COP trajectory as the consequence of velocity-based control. Our results indicated slightly diffusive velocity dynamics, close to Brownian motion, over the short term. Basically, this dynamics can be modeled by a first-order autoregressive process including a constant that induces a linear trend in the series:
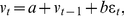
(8)where *v_t_* represents velocity at time *t*, *a* is a constant and *ε_t_* a white noise of strength *b*.

As shown experimentally, the long-term dynamics of COP velocity is anti-persistent: the evolution in velocity reverses its direction when it reaches an upper or lower limit, *i.e.*, when the absolute velocity value exceeds a given threshold *T* (see Figure 4). This dynamics can be obtained by changing the sign of *a* each time that velocity reaches the upper or the lower limit. For simplicity, we consider that these limits are symmetrically positioned at +/−*T*.

This equation yields series that reproduce the expected to and fro of velocity between the two boundary values, but in an excessively systematic manner. More realistic dynamics can be obtained by making the linear trend of Equation 8 dependent on the current absolute velocity (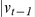):
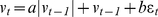
(9)


According to this equation, the higher the absolute velocity, the higher the contribution of the linear trend to its dynamics. Note that the goal of Equation 9 was just to mimic the short-term behavior of velocity as closely as possible. We had no specific assumptions about possible correspondences between the terms included in the model and the neurophysiological processes involved in postural control. Our aim was simply to check whether the bounding of velocity, imposed on this short-term dynamics, would allow us to simulate the empirically observed dynamics, for the velocity and position series.

We simulated 100 series of 1024 points using Equation 9, with *T* = 10, *a* = 0.16, and *b* = 1.6. These values were chosen for approximately fitting the shape of the empirical series. The corresponding position series were computed by integrating the velocity series. We analyzed the simulated position and velocity series using DFA and PSD. Figure 5 shows an example of the series of position and velocity obtained. Figure 6 presents the average diffusion plots and power spectra obtained from 26 randomly chosen simulated series. In all cases, the graphical signatures of the simulated series were similar to those obtained from the experimental series. For the position series, the DFA yielded a mean (*N* = 100) slope of 1.77 (±0.02) in the short term and 1.25 (±0.51) in the long term. For the velocity series, the mean slopes were 1.38 (±0.03) in the short term and 0.35 (±0.17) in the long term. When applied to the position series, the spectral analysis yielded a mean slope of −3.53 (±0.10) in the high-frequency region (short-term) and −1.63 (±0.94) in the low-frequency region (long-term). For the velocity series, the spectral slopes were of −1.84 (±0.10) in the high frequencies, and 0.80 (±0.74) in the low frequencies.
